# Atrial Fibrillation in Heart Failure Is Associated with High Levels of Circulating microRNA-199a-5p and 22–5p and a Defective Regulation of Intracellular Calcium and Cell-to-Cell Communication

**DOI:** 10.3390/ijms221910377

**Published:** 2021-09-26

**Authors:** Anna Garcia-Elias, Marta Tajes, Laia Yañez-Bisbe, Cristina Enjuanes, Josep Comín-Colet, Selma A. Serra, José M. Fernández-Fernández, Kathryn W. Aguilar-Agon, Svetlana Reilly, Julio Martí-Almor, Begoña Benito

**Affiliations:** 1 Vascular Biology and Metabolism Program, Vall d’Hebron Research Institute (VHIR), Passeig de la Vall d′Hebron 119-129, 08035 Barcelona, Spain; laiayb19@gmail.com; 2 Group of Biomedical Research in Heart Diseases, Hospital del Mar Medical Research Institute (IMIM), Doctor Aiguader 88, 08003 Barcelona, Spain; cicelyflyn@gmail.com (M.T.); jmartialmor@gmail.com (J.M.-A.); 3 Research Group in Cardiovascular Disorders (BIOHEART), Bellvitge Biomedical Research Institute (IDIBELL), Avinguda de la Granvia de l’Hospitalet, 199, 08908 L′Hospitalet de Llobregat, Spain; cristinaenjuanes@gmail.com (C.E.); josepcomin@gmail.com (J.C.-C.); 4 Cardiology Department, Hospital de Bellvitge, Carrer de la Feixa Llarga s/n, 08907 L’Hospitalet de Llobregat, Spain; 5 Department of Clinical Sciences, University of Barcelona School of Medicine, 08036 Barcelona, Spain; 6 Laboratory of Molecular Physiology, Department of Experimental and Health Sciences, Universitat Pompeu Fabra, Doctor Aiguader 88, 08003 Barcelona, Spain; selmaserra@gmail.com (S.A.S.); jmanuel.fernandez@upf.edu (J.M.F.-F.); 7 Radcliffe Department of Medicine, Division of Cardiovascular Medicine, John Radcliffe Hospital, University of Oxford, Oxford OX3 9DU, UK; kathryn.aguilar-agon@cardiov.ox.ac.uk (K.W.A.-A.); svetlanareilly@aol.com (S.R.); 8 Cardiology Department, Hospital del Mar. Passeig de la Barceloneta 25, 08003 Barcelona, Spain; 9 Department of Medicine, Universitat Autònoma de Barcelona, 08035 Barcelona, Spain; 10 Cardiology Department, Hospital Universitari Vall d’Hebron, Universitat Autonoma Barcelona, Passeig de la Vall d′Hebron 119-129, 08035 Barcelona, Spain; 11 Centro de Investigación Biomédica en Red de Enfermedades Cardiovasculares (CIBER-CV), Instituto de Salud Carlos III, 28029 Madrid, Spain

**Keywords:** heart failure, atrial fibrillation, atrial remodeling, microRNA, biomarkers, HL-1 cells, calcium regulation, L-type calcium channels, connexin 40, NCX1

## Abstract

MicroRNAs (miRNAs) participate in atrial remodeling and atrial fibrillation (AF) promotion. We determined the circulating miRNA profile in patients with AF and heart failure with reduced ejection fraction (HFrEF), and its potential role in promoting the arrhythmia. In plasma of 98 patients with HFrEF (49 with AF and 49 in sinus rhythm, SR), differential miRNA expression was determined by high-throughput microarray analysis followed by replication of selected candidates. Validated miRNAs were determined in human atrial samples, and potential arrhythmogenic mechanisms studied in HL-1 cells. Circulating miR-199a-5p and miR-22-5p were significantly increased in HFrEF patients with AF versus those with HFrEF in SR. Both miRNAs, but particularly miR-199a-5p, were increased in atrial samples of patients with AF. Overexpression of both miRNAs in HL-1 cells resulted in decreased protein levels of L-type Ca^2+^ channel, NCX and connexin-40, leading to lower basal intracellular Ca^2+^ levels, fewer inward currents, a moderate reduction in Ca^2+^ buffering post-caffeine exposure, and a deficient cell-to-cell communication. In conclusion, circulating miR-199a-5p and miR-22-5p are higher in HFrEF patients with AF, with similar findings in human atrial samples of AF patients. Cells exposed to both miRNAs exhibited altered Ca^2+^ handling and defective cell-to-cell communication, both findings being potential arrhythmogenic mechanisms.

## 1. Introduction

Heart failure (HF), affecting 1–2% of the adult population and up to > 10% of individuals over 70 years, is the cardiovascular disorder associated with the highest morbidity and mortality rates, particularly in the subgroup of patients with reduced ejection fraction [1]. Atrial fibrillation (AF) occurs in 25–40% of patients with HF and reduced ejection fraction (HFrEF) [2]. The coexistence of both conditions is facilitated by the common pathogenic mechanisms shared between HF and AF, which promote one another [3,4]. For example, impaired regulation of intracellular Ca^2+^, electrical remodeling and the development of fibrosis are seen in the atria of AF patients and in the ventricles of failing hearts [3,4]. These mechanisms favor atrial arrhythmia susceptibility and impaired ventricular contraction [4].

The presence of AF in HF patients has serious prognostic implications. In patients with HFrEF, a preexisting AF increases the risk of cardiovascular death or hospital readmission by more than 20% at a three-year follow-up [5]. Moreover, the development of new-onset AF in patients with HF has been associated with doubled mortality in follow-up [5,6,7]. These observations emphasize the need to treat and prevent the occurrence of AF in patients with HF.

Defining the mechanisms underlying AF occurrence and progression in HF patients is critical for the development of new treatment tools. Despite sharing similar cardiac substrates, AF requires the presence of an electrical trigger to generate the arrhythmia, an element that is not obligatory in HF [8,9]. It is plausible that the presence of AF in the setting of HF is accompanied by certain mechanistic features, related to electrical or structural remodeling, that are specific to this condition and are differential to those that are present in HF without concomitant AF.

During the last decade, microRNAs (miRNAs), 21 to 25 nucleotides sequences that regulate gene expression, have been implicated in the pathogenesis of numerous diseases [10]. In the setting of HF and AF, a number of miRNAs like miR-21, miR-30, miR-31, miR-133 and miR-29 have been shown to mediate structural remodeling, an important mechanism underlying both ventricular dysfunction and the occurrence of AF [11,12,13,14]. The presence of miRNAs in circulation makes them attractive as potential biomarkers of disease [15]. For example, use of multi-miRNA panels in conjunction with the levels of NT-proBNP has been found to improve diagnostic accuracy of HF [16]. Similarly, a particular miRNAs signature in blood, characterized by increased expression of miRNA-103a, -107, -320d, -486, andlet-7b, has also been related to the presence of persistent but not paroxysmal AF [17]. While previous reports focused majorly on the role of selected miRNAs [13,18,19], instances of unbiased profiling of circulating miRNA using high-throughput screenings in patients with combined HF and AF are scarce.

Here, we tested whether the presence of AF in patients with HFrEF would be associated with a distinct systemic miRNA profile in plasma, which could be reflective of the particular mechanistic pathways altered when both entities co-exist. We first characterized the circulating miRNA profiles of two very homogeneous populations of HFrEF patients with and without AF, following a two-phase approach of screening and replication. We observed increase of miR-199a-5p and miR-22-5p in plasma of patients with AF + HFrEF compared to those with HFrEF without the arrhythmia, and in the atrial tissue obtained from patients with AF. HL-1 cells transfected with both miRNAs had altered Ca^2+^ handling that correlated with decreased expression and functionality of L-type calcium channel (LTCC) and NCX1, and defective cell-to-cell communication due to a decrease in connexin 40 (Cx40). Our findings, therefore, identify significant upregulation of circulating levels of miR-22-5p and miR-199a-5p in patients with HFrEF and AF that may potentially facilitate the promotion of the arrhythmia in this setting.

## 2. Results

### 2.1. HFrEF Patients with AF Have Higher Plasmatic Levels of miRNA-199a-5p and miRNA-22-5p

The clinical characteristics of patients included in the discovery phase are shown in Supplementary File, Table S1. Briefly, 55% were men and the mean age was 75 in both groups. The prevalence of cardiovascular risk factors and comorbidities was high (Table S1). Mean left ventricular ejection fraction (LVEF) was around 30%, and New York Heart Association (NYHA) class was similar between patients in permanent AF (permAF, including long-standing persistent and permanent AF) and those in sinus rhythm (SR). In this population, the microarray study revealed 76 miRNAs that were differentially expressed between both groups. Based on their higher statistical significance, plasma stability or clinical relevance, 18 were selected for replication (Supplementary File, Table S2).

Characteristics of patients included in the replication cohort are shown in Table 1. Mean age was 73 for both groups, and 77% were men. BMI was higher in patients with permAF. Prevalence of hypertension, diabetes and CKD was high in both groups. HF status was similar in patients with permAF and SR, with a mean LVEF 32% and no significant differences in treatment, although patients with permAF tended to present more advanced NYHA class (Table 1).

The results of the replication study are summarized in Table 2. Two microRNAs (miR-199a-5p and miR-22-5p) remained differentially expressed between groups, showing 1.92-fold and 1.55-increased expression in permAF versus SR, respectively.

### 2.2. MiR-199a-5p and 22–5p Expression Levels Are Higher in Atrial Samples of AF Patients

**Figure 1:**
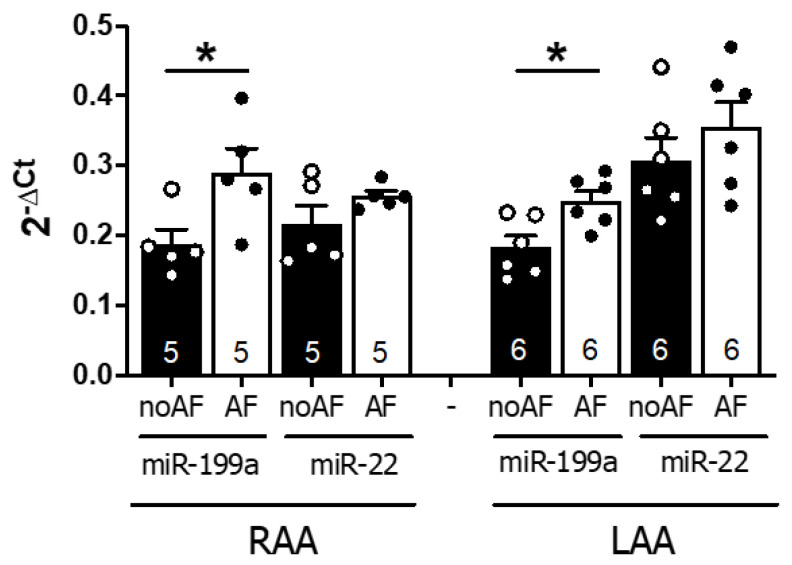
MicroRNA determination in human left and right atrial appendages. Expression levels of miR-199a-5p and miR-22-5p of AF patients (white columns) compared to those in sinus rhythm (no-AF, black columns). Scatter dot blots represent each individual patient, and the number within the column represents the total of independent experiments performed. * *p* < 0.05, RAA: right atrial appendage, LAA: left atrial appendage.

To assess whether circulating levels of miR-199a-5p and miR-22-5p correlated with the atrial levels of both miRNAs, we assessed miRNA expression in human right and left atrial appendages obtained from 22 patients with AF or SR (no AF) referred for elective cardiac surgery (Supplementary File, Table S3 for clinical characteristics). As shown in Figure 1, miR-199-5p expression levels were increased in both RAA (*p* = 0.034) and LAA (*p* = 0.014) in AF versus no AF patients, whereas miR-22-5p showed a similar but non-significant trend.

These observations indicate that local overexpression of miR-199a-5p and miR-22-5p is associated with AF, and suggest that the increase in atrial miRNA levels may, in part, contribute to the elevated systemic levels observed in AF.

### 2.3. Overexpression of miR-199a-5p and miR-22-5p Is Associated with Reduced Expression of Proteins Involved in Ca^2+^ Homeostasis and Cell-to-Cell Communication

Since circulating expression of both miRNAs was confirmed in atrial samples of AF patients, we sought to evaluate the potential role of miR-199a-5p and miR-22-5p in AF promotion by studying the effects of simultaneous overexpression of both miRNAs (miR-199a+miR-22) in atria-derived HL-1 cardiomyocytes versus cells transfected with miR-negative control (miR-NC). The efficiency of miRNA transfection was assessed by qPCR (Supplementary File, Figure S1).

**Figure 2:**
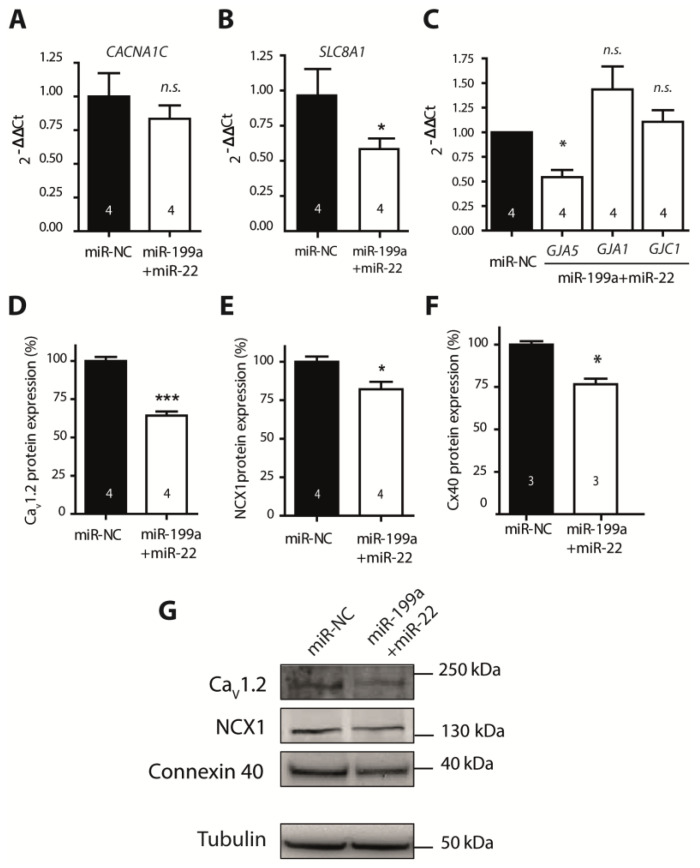
Gene and protein expression (**A**–**C**). Expression levels of *CACNA1C*, *SLC8A1* and the connexin members, *GJA5*, *GJA1* and *GJC1* in HL-1 cells transfected with miR-NC (black columns) or miR-199a+miR-22 (white columns). Normalized to miR-NC (**D**–**F**). Protein expression levels of Cav1.2, NCX1 and Cx40, normalized to the endogenous control, tubulin (**G**). Representative WB experiments in HL-1 cells transfected with miR-22+199a and miR-NC. All proteins (Cav1.2, NCX1, connexin 40 and tubulin) were blotted on the same gel. This is a representative image of four independent experiments. Numbers within columns represent the number of independent experiments done. * *p* < 0.05, *** *p* < 0.0001, n.s.: non-significant.

Among all predicted targets for both miRNAs (TargetScan), we focused on those molecules involved in Ca^2+^ homeostasis and cell-to-cell communication, key elements in AF pathophysiology [20,21]. We first evaluated the expression of the main target genes involved in both pathways in miR-199a+miR-22 and miR-NC cells. Results are summarized in Figure 2 and Figure S3 (Supplementary File). Gene expression of *CACNA1C* (encoding the alpha-subunit of the LTCC, Figure 2A) showed a trend, though it did not reach statistical significance, towards a reduction in miR-199a+miR-22 HL-1 cells compared to miR-NC. Western blot (WB) revealed a significant 30% reduction in Cav1.2 expression (Figure 2D,G, *p* < 0.0001). Gene expression of *SLC8A1* (encoding NCX) was significantly reduced in miR-199a+miR-22 compared to miR-NC cells (Figure 2B), which translated into a 20% reduction in the protein expression (Figure 2E,G). Gene expression of RYR2, encoding the ryanodine receptor, was reduced in miR-199a+miR-22 cells, with no change in ATP2A2, PLN, or CAMK2A (encoding SERCA2, phospholamban and the alpha-subunit of the calcium-calmodulin-dependent kinase II, respectively (Supplementary File, Figure S3).

The expression studies of the three main connexins in the heart revealed that only *GJA5*, encoding Cx40, the main connexin at the atrial level, was significantly reduced in miR-199a+miR-22 compared to miR-NC cells (*p* = 0.029, Figure 2C), with no significant changes in the genes encoding Cx43 and Cx45 (GJA1 and GJC1, respectively). Similarly, protein expression of Cx40 was decreased by 25% in cells transfected with miR-199a+miR-22 (*p* = 0.0036, Figure 2F,G).

### 2.4. Overexpression of miR-199a-5p and miR-22-5p Is Associated with Reduced Intracellular Ca^2+^ Levels and a Decrease in Barium Currents (I_Ba_)

**Figure 3:**
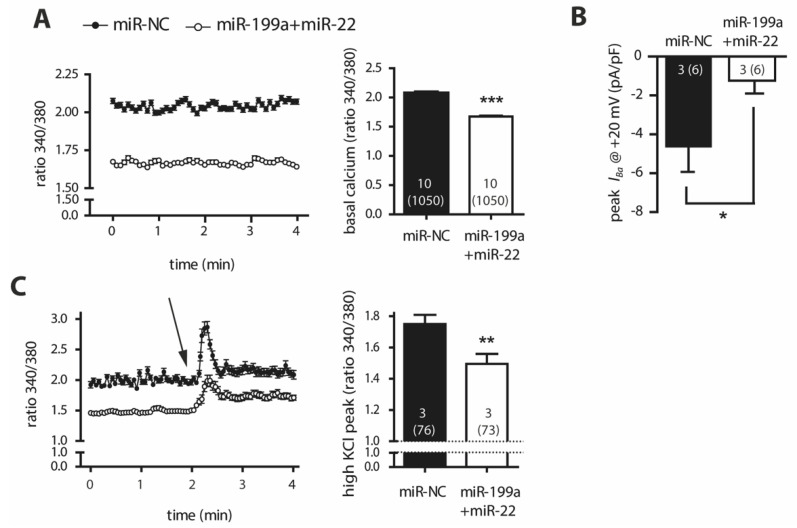
Calcium entrance through LTCC and cytosolic recordings in HL-1 cells transfected with miR-199a+miR-22 (white) or miR-NC (black) (**A**). Left panel, fluctuations of intracellular calcium recorded in basal conditions in cells loaded with Fura-2AM. Right panel, calcium levels graphed and averaged in column bars (**B**). Ba^2+^ peak current through Cav1.2 channels at +20 mV recorded using patch-clamp technique in whole-cell configuration (**C**). Left panel, intracellular calcium fluctuations under depolarizing conditions induced by the application of an extracellular solution containing 75 mM KCl (arrow). Right panel, maximum calcium levels graphed and averaged in column bars. * *p* < 0.05, ** *p* < 0.005, *** *p* < 0.0001. Numbers within the columns represent the number of independent experiments performed with the total number of cells analyzed in parenthesis.

To investigate the functional consequences of the decrease of Cav1.2, we monitored the intracellular calcium measurements under resting conditions, which revealed a prominent reduction in cytosolic Ca^2+^ levels of miR-199a+miR-22 HL-1 cells compared to miR-NC (*p* < 0.0001, Figure 3A). Patch-clamp experiments showed a marked reduction in *I_Ba_*, the surrogate for the Ca^2+^ currents, in miR-199a+miR-22 HL-1 cells compared to miR-NC (*p* = 0.026, Figure 3B). Calcium influx through Cav1.2 was also impaired in miR-199a+miR-22 HL-1 cells under depolarizing conditions induced by high extracellular potassium levels (Figure 3C, *p* = 0.004). These findings, together with the fact that no differences were observed in the fast-inactivation kinetics nor in cell capacitance between miR-199a+miR-22 and miR-NC HL-1 cells (Supplementary File, Figure S2), suggest that the reduction of inward currents present in miR-199a+miR-22 HL-1 cells is due to a decrease in the total protein content of channel rather than a functional defect.

### 2.5. Cells Transfected with miR-199a-5p and miR-22-5p Have Higher Ca^2+^ Levels in the Sarcoplasmic Reticulum and a Slower Caffeine-Induced Ca^2+^ Decay

**Figure 4:**
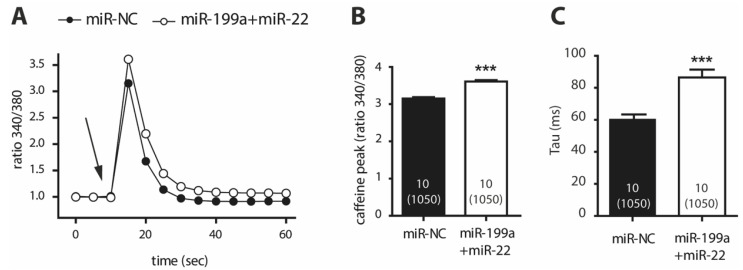
Caffeine-induced calcium response in HL-1 cells transfected with miR-199a+miR-22 (white) or miR-NC (black) (**A**). Normalized intracellular calcium fluctuations with the application of 10 mM caffeine (arrow). To assess caffeine-induced maximum peak, basal calcium levels in both transfection conditions were normalized to one (**B**). Maximum calcium levels post-caffeine graphed and averaged in column bars (**C**). Calcium decay expressed as the time constant, Tau. *** *p* < 0.0001. Number within the column represents the number of independent experiments performed with the total number of cells analyzed in parenthesis.

Given the reduced expression of NCX1, we evaluated the rapid Ca^2+^ buffering after a caffeine-induced response, driven by the SERCA2 pump at the sarcoplasmic reticulum (SR) and NCX1 at the cytosolic membrane. Figure 4A represents the dynamics of Ca^2+^ release in response to 10 mM caffeine in miR-199a+miR-22 and miR-NC cells after normalizing to basal Ca^2+^ levels. An increase in the peak of the caffeine-induced Ca^2+^ release was observed in miR-199a+miR-22 HL-1 cells (Figure 4B), suggesting higher SR Ca^2+^ content in transfected cells. More importantly, the decay of Ca^2+^ release (calculated from the time constant, Tau) was slower in miR-199a+miR-22 cells compared to miR-NC cells (Figure 4C, *p* < 0.0001). This response was observed in the presence of decreased gene and protein expression of NCX1 and no changes in gene expression of *ATP2A2*, as shown previously (Figure 2 and Supplementary File, Figure S3).

Taken together, these results indicate that upregulation of miR-199a and miR-22 in HL-1 cells activates Ca^2+^ release in response to caffeine (most likely due to increased Ca^2+^ content in the sarcoplasmic reticulum) and impairs Ca^2+^ buffering (primarily via downregulation of NCX1, a predicted target of both miR-199a-5p and miR-22-5p). Both mechanisms may contribute to the overall reduced cytosolic levels of Ca^2+^, as a potential compensatory response.

### 2.6. Overexpression of miR-199a-5p and miR-22-5p Is Associated with a Reduction in Cx40 and Impaired Cell-to-Cell Communication

**Figure 5:**
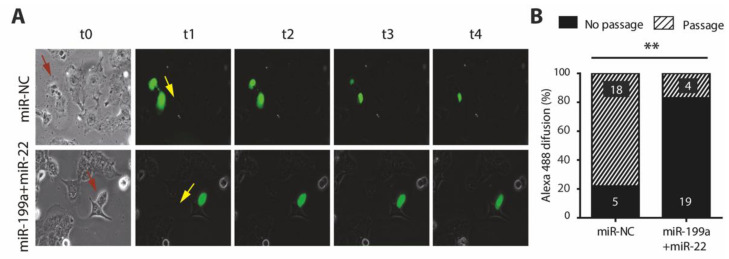
Cell-to-cell communication in HL-1 cells transfected with miR-199a+miR-22 (white) or miR-NC (black) (**A**). A representative experiment following the pass of Alexa 488 between two cells (pairs seen in the left bright field image with the injected cell marked with a red arrow, and the expected direction of Alexa flow is marked with a yellow arrow). Images were taken at four different time points over the course of 90 min (t1–t4). The passage through these cells was so fast that even at the beginning of the recording (t1) the fluorescent dye was already present in both cells (**B**). Percentage of cells that allowed (stripped) or did not allow (plain) passage of Alexa 488 to neighboring cell. Numbers within the columns represent the absolute value of cells, with a total of 23 in both cases. ** *p* < 0.005.

While connexins have been consistently implicated in AF promotion, Cx40 and Cx45 are predicted targets of miR-199a-5p and miR-22-5p, respectively. As shown previously (Figure 2), only *GJA5* and Cx40 protein expression were significantly reduced in miR-199a+miR-22 compared to miR-NC cells. A functional consequence of these findings was assessed using HL-1 transfected cells at very low confluence, and monitoring the diffusion of a small fluorescent dye (Alexa 488) through connexins between two contiguous cells. A total of 23 paired cells of each transfection condition (miR-199a+miR-22 and miR-NC) were analyzed from three independent experiments. Images from a representative experiment of each condition are shown in Figure 5A. Passage of Alexa 488 to the neighboring cell was seen in 4 (17%) of miR-199a+miR-22 HL-1 cells, and in 18 out of 23 (78%) of the miR-NC cells (Figure 5B). These results confirm a defective cell-to-cell communication in miR-199a+miR-22 cells, possibly explained by a reduction in Cx40, providing a mechanism that could potentially contribute to AF promotion.

## 3. Discussion

The presence of AF entails a worse outcome in patients with HF [5,6,7]. Thus, studying molecular mechanisms contributing to the development of the arrhythmia in this setting is the focus of intense research. Our study provides new insights on one potentially important mechanism. We demonstrated an association of high circulating levels of miR-199a-5p and miR-22-5p with the presence of AF in HFrEF patients. Both miRNAs, especially the former, were increased in atrial samples of AF patients, indicating that these miRNAs could have a cardiac origin and be directly involved in AF promotion.

Although previous studies have described an association of altered circulating miRNA profile with AF, almost all of them excluded HF patients [15,17]. Very few reports have explored the joint effect of both conditions on miRNA levels, and these have primarily focused on the role of selected miRNAs. For example, Goren et al. found a reduction in miR-150 in platelets of patients with both AF and HF [18] and Wei et al. reported lower serum levels of miR-126 when both disorders coexist [19]. Furthermore, Dawson et al. found that miR-29b plasma levels were decreased in patients with either HF or AF, and were further decreased in patients with both HF and AF [13]. To date, an unbiased high-throughput screening of miRNA profile has not been performed.

In our study, using high-throughput technology, we found that circulating levels of miR-199a-5p and miR-22-5p were significantly increased in patients with AF. Although our results did not reproduce those of Goren, Wei or Dawson, possibly due to the use of different methods for miRNA profiling and the study of small populations with different genetic backgrounds [22,23], both miR-22-5p and miR-199a-5p have been linked to cardiac remodeling in HF or AF. Both were reported to be upregulated in the right appendage samples obtained from patients undergoing cardiac surgery with AF compared to those without AF [24]. MiR-22-5p has been associated with HF [25] and was suggested to serve as a key regulator of cardiac hypertrophy and remodeling [26]. MiR-199a-5p has been associated with AF in diverse contexts. Two independent groups reported increased miR-199a-5p expression in atrial tissue of AF patients [24,27], which was associated with a decrease in FKBP5, a possible contributor to the pro-arrhythmic substrate. By contrast, another study observed a downregulation of miR-199a in atrial samples of patients undergoing cardiac surgery who later developed post-operative AF [28], although this is a specific setting with the mechanistic features characteristic of a short-standing self-limiting AF episode that might not be applicable to all types of AF.

Importantly, we found a significant increase in the atrial tissue content of miR-199a-5p in the presence of long-standing AF, and a clear, although not statistically significant, trend towards a reduction of miR-22-5p levels. The latter finding could be due to the fact that patients in the atrial sub-study did not have HF. However, the overall results suggest that increased circulating levels of both miR-199a-5p and miR-22-5p could, at least in part, be related to the upregulated miRNA expression in the atrial myocardium. Thus, our results support the role of miR-199a-5p and miR-22-5p as circulating biomarkers potentially linked to the pathophysiology of the arrhythmia.

To understand functional consequences of upregulated miR-199a-5p and miR-22-5p in atrial tissue, we explored some potential mechanisms by which both miRNAs could participate in AF promotion. Overexpression of miR-199a and miR-22 in cultured HL-1 cells caused a reduction in resting cytoplasmic Ca^2+^ concentrations, decreased protein levels of Cav1.2, and a marked reduction of *I_Ba_*. Since protein levels were only decreased by 20% whereas currents were almost abolished in the patched cells, we cannot exclude that these miRNAs may alter protein function not only by lowering its expression but also by altering its trafficking and/or the protein surface localization. In agreement with this, it has been shown that miR-199a can strongly influence intracellular trafficking by regulating genes that coordinate retrograde transport and endocytosis [29,30].

Both the decrease in Cav1.2 and *I_CaL_* are implicated in AF pathogenesis, through shortening of action potential duration that promotes re-entry circuits [31,32,33]. Cells transfected with miR-199a+miR-22 also exhibited an increased response to caffeine, indicative of a greater Ca^2+^ content in the sarcoplasmic reticulum. In line with our results, Gurha et al. reported that the miR-22-5p knock-out mice exhibited a reduction in the sarcoplasmic reticulum Ca^2+^ content [25]. The intrinsic molecular mechanisms were not investigated in detail in this study, but an initial approach evidenced lower mRNA expression of *RYR2* and unchanged expression of *ATP2A2, PLN* and *CAMK2A* genes. Finally, we found decreased expression and function of NCX1 in miR-199a+miR-22 transfected cells. Previous reports found that AF is associated with increased NCX1 causing delayed afterdepolarizations (established triggers of the arrhythmia) [32,34]. However, our finding might argue for a compensatory mechanism of the reduced cytoplasmic Ca^2+^ levels seen in miR-199a+miR-22 cells rather than a mechanism participating in the promotion of the arrhythmia. It is worth mentioning, however, that in a murine transverse aortic constriction model, reduced NCX activity was associated with the development of HF [35]. Although speculative at this stage, higher abundance of miR-199a and miR-22-5p in our HFrEF + AF patients might trigger decrease of NCX1 levels which in turn may contribute to a HF substrate, lining up with the theory of the vicious electromechanical circle between both entities [3].

Overexpression of miR-199a+miR-22 in HL1 cells also caused a 20% reduction in the protein levels of Cx40 and impaired cell-to-cell communication in 80% of cells. Connexins are hemichannels that need to couple with one another to form functional gap-junctions between neighboring cells. Specifically, Cx40 dysfunction as well as polymorphisms and mutations in this protein at the cardiac level has been associated with AF [36,37,38]. In our study, the miRNA overexpression in cultured HL-1 cells may have reduced the probability of finding paired cells with sufficient expression of Cx40 forming complete gap-junctions. This could explain why the overall effects on communication seem larger compared to only a moderate decrease in the protein.

Our study has some limitations to consider. First, we studied the effects of two preselected miRNAs, found to be significantly increased in plasma of patients with HFrEF and AF, on cultured cardiac cells. We cannot exclude that these circulating miRNAs do not have their main target at the heart, or have other systemic effects, or even be secondary (and not causative) to the particular cardiac remodeling present in patients with HFrEF and AF. However, both miR-199a-5p and miR-22-5p have been previously implicated in AF pathophysiology and were found to be upregulated in atrial samples of patients with AF in our study and others [24,27]. Importantly, we only studied the miRNA profile in patients with AF and HFrEF. Whether our findings are applicable for patients with AF and HF with mid-range or preserved LVEF remains to be addressed in future studies. Persistent SR could only be confirmed in 21.4% of patients who had an intracardiac electronic device implanted and thus were continuously monitored. We cannot exclude that some subclinical paroxysmal AF episodes might have occurred in the remaining patients, who on the other hand were monitored by repeated ECGs at 6-month intervals. Another limitation is the use of an in vitro overexpression system, which inevitably introduces an experimental bias due to the supraphysiological increase in miRNA levels (Supplementary File, Figure S1), and this could potentially overestimate the effects of miRNAs on Ca^2+^ dynamics and cell-to-cell communication. The use of miRNA inhibitors could be a safer alternative to analyze the biologically relevant outcomes. Building on this, the in vitro experiments were performed with overexpression of both miRNAs simultaneously. We chose this approach because the aim of our project was to mimic the overall miRNA profile that was upregulated in patients with AF and HFrEF and its potential effects on arrhythmia promotion. The effects of overexpression of individual miRNAs were not explored in an attempt to keep the potential cellular interactions between both miRNAs. Finally, we mainly focused our mechanistic study on LTCC, NCX and Cx-40, because, among all predicted targets for both miRNAs that could be involved in Ca^2+^ homeostasis and cell-to-cell communication, expression of these genes and proteins was significantly altered in miR-199+miR-22 transfected cells. We cannot exclude that other pro-arrhythmic mechanisms might be also triggered by miR-199-5p and miR-22-5p. Yet, the aim of this work was to evaluate whether there were differential patterns of circulating miRNAs in HFrEF patients with and without AF, and whether these differences could contribute to our understanding of the mechanisms promoting the arrhythmia.

## 4. Materials and Methods

### 4.1. Human Plasma Study

#### 4.1.1. Study Population

Patients were selected from the DAMOCLES registry (Definition of the neuro-hormonal Activation, Myocardial function, genOmic expression and CLinical outcomes of hEart failure patients), a HFrEF cohort collected prospectively at Hospital del Mar (Barcelona, Spain) between 2004 and 2013 [39,40]. For the purpose of the present study, only patients with HFrEF fulfilling the ESC guidelines criteria (i.e., with symptoms ± signs of HF and a LVEF <40%) were included [41]. All patients were engaged into a hospital/primary care integrated chronic management program of HF, with weekly tele-monitoring nurse-based follow-up visits and regular on-site visits every 6 months, or earlier if appropriate. Exclusion criteria were: significant primary valvular disease, clinical signs of fluid overload, pericardial disease, restrictive cardiomyopathy, hypertrophic cardiomyopathy, hemoglobin (Hb) levels <8.5 g/dL, active malignancy, and chronic liver disease.

To clearly stratify patients according to the presence of AF, only those with long persistent (>1 year) or permanent AF (permAF), or otherwise those with no previous history of AF (sinus rhythm, SR), were included. SR patients were required to be in permanent SR if they had any intracardiac electronic device implanted or to be persistently found in SR in all on-site follow-up visits. Patients with other forms of AF were excluded from the study.

Blood was collected from all patients in stable clinical status and immediately centrifuged at 1550× *g* for 10 min. Plasma was transferred to RNase/DNAse-free tubes and stored at −80 °C until use. The study was conducted in accordance with the Declaration of Helsinki, and approved by the local Ethics Committee. All patients gave written informed consent.

#### 4.1.2. Experimental Design

**Figure 6:**
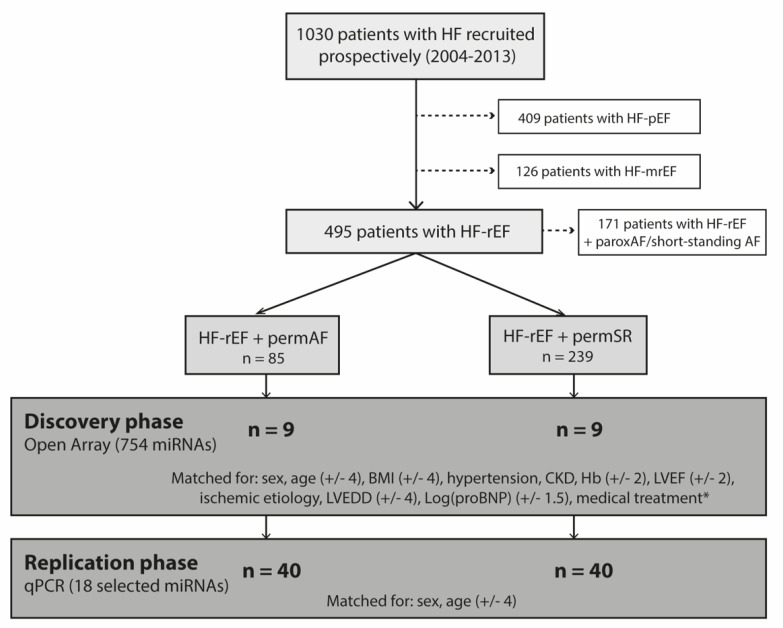
Study design. Of 495 patients with HFrEF, 18 (9 with permAF and 9 with SR) were selected for the discovery phase, and 80 (40 per group) for the replication phase. Matching variables for each phase are presented, with the accepted range in parenthesis. Abbreviations: HF-pEF, mrEF, and rEF: heart failure with preserved (pEF), mid-range (mrEF) and reduced ejection fraction (rEF), respectively, according to the ESC guidelines; (41) permAF: permanent AF; SR: permanent sinus rhythm (no history of past or present AF); BMI: body mass index; CKD: chronic kidney disease; Hb: hemoglobin; LVEF: left ventricular ejection fraction; LVEDD: left ventricular end-diastolic diameter; Log(proBNP): pro-BNP values at baseline in Log expression; * included matching for treatment with betablockers, angiotensin-converting-enzyme inhibitors (ACEI), angiotensin-receptor blockers (ARB), and diuretics.

This was a case-control study in which the miRNA expression of HFrEF patients with permAF (cases) was compared to that of HFrEF and SR (controls) following a two-step approach: (1) discovery phase using microarray data; (2) replication phase of those miRNAs showing the greatest association in the microarray study. For the discovery phase, a total of 18 patients (9 per group) were selected, matched for sex, age, body mass index and HF parameters in order to diminish confounding factors and guarantee that the populations of study were very similar except for the presence of AF. For the replication phase, we selected an independent cohort of 80 patients (40 per group) matched exclusively for age and sex, accepting the potential interference of confounding factors in an attempt to prove the association of biomarkers with AF irrespective to other baseline clinical conditions. The experimental design of the study is summarized in Figure 6.

### 4.1.3. MicroRNA Extraction and Profiling

MiRNAs were isolated from plasma using the miRNeasy Serum/Plasma kit (Qiagen, Hilden, Germany), and retrotranscribed into cDNA with the TaqMan Advanced miRNA cDNA Synthesis Kit. In the discovery phase, an OpenArray^®^ technology was used to screen 754 of the most expressed human miRNAs. Expression levels of all miRNAs were analyzed with the Cloud software and the mean global normalization was used. Those miRNAs found to have a significant differential expression among groups (*p* < 0.05 and fold change >2 or <0.5) were selected for replication. MiRNA determination in the replication phase was performed by qPCR using the Fast Advanced master mix with specific TaqMan^®^ miRNA probes (Life Technologies). MiR-660, miR-92a and miR-10b showed high stability between groups, and were therefore used as controls for normalization. Results were analyzed using the Expression Suite software. All reagents and sequencing technologies were from ThermoFisher, Waltham, MA, USA.

## 4.2. Human Atrial Samples

### 4.2.1. Cardiac Tissue Collection

Whole-tissue homogenates from samples of the right and left atrial appendages (RAA, LAA) were obtained from patients in AF or SR (no AF) who were admitted for the first-time elective on-pump cardiac surgery in the John Radcliffe Hospital (Oxford, UK). Tissues were quickly rinsed of blood and snap-frozen until experimental use. Experimental work in human specimens was approved by the local Research Ethics Committees (REF #18/SC/0404) and all patients gave informed written consent.

### 4.2.2. MicroRNA Profiling

Expression levels of hsa-miR-199a-5p and hsa-miR-22-5p transcripts in human RAA and LAA were measured by qPCR. Total RNA was extracted using the mirVana Kit (Applied Biosystems, Waltham, MA, USA) according to the manufacturer’s instructions. Quantitative PCR was carried out using the TaqMan Advanced miRNA cDNA synthesis kit, TaqMan Advanced miRNA stem-loop primers (Supplementary File, Table S4), and TaqMan Universal Master Mix (all from Applied Biosystems, Waltham, MA, USA). Each reaction was performed in duplicate using the QuantStudio 7 Flex Real-Time PCR system (Applied Biosystems, Waltham, MA, USA) using miRNA stem-loop primers (Table S1), and TaqMan Universal Master Mix. Relative quantification was calculated using the comparative threshold cycle method (2^−^^△Ct^); the expression of the target was normalized to the mean of the two housekeeping genes, hsa-miR-191-5p and hsa-miR-26a-5p.

## 4.3. Molecular Studies

### 4.3.1. Cell Cultures and Transfection

Atrial HL-1 cardiomyocytes were cultured and maintained in Claycomb medium (Sigma Aldrich, Sain Louis, MO, USA) supplemented with 10% FBS, 1% penicillin/streptomycin, 1% norepinephrine and 1% glutamine. They were transfected using lipofectamine RNAiMAX with 30 nM of mimic mmu-miR-199a-5p (assay MC10893) and 30 nM of miRNA mimic mmu-miR-22-5p (assay MC11752), in the text, miR-199a+miR-22, or with 60 nM of mirVana™ miRNA Mimic Negative Control #1, in the text, miR-NC. Experiments were performed 24 h after transfection. Transfection reagents were all from ThermoFisher, Waltham, MA, USA.

#### 4.3.2. qPCR and Western Blot Analyses

To evaluate gene expression, RNA was extracted using the Nucleospin^®^ RNA extraction kit (Macherey-Nagel, Duren, Germany). RNA was retro-transcribed into cDNA and the qPCR was performed using the specific TaqMan probes cycled in a 7500 ThermoCycler (Applied Biosystems, Waltham, MA, USA). HPRT was used as the endogenous control. Each transfection condition was repeated in 4 independent experiments. Expression level measurements are expressed as the mean ± standard error of the mean (SEM) of the 4 results.

For protein determination, total protein was extracted from HL-1 cell cultures. In each lane, 25 μg of protein was loaded and separated on 4–12% SDS-PAGE gels before transferring to a nitrocellulose membrane. Membranes were blocked for 1 h at room temperature with TBST buffer containing 5% non-fat milk and incubated overnight at 4 °C with the following primary antibodies, all at a 1/1000 dilution: anti-Cav1.2 (L-type calcium channel alpha subunit, ACC-003, Alomone Labs, Jerusalem, Israel), anti-NCX1 (ANX-011, Alomone Labs, Jerusalem, Israel), anti-Cx40 (36–4900, Thermo Fisher Scientific, Waltham, MA, USA) and anti-beta-tubulin antibody (ab6046, Abcam, Cambridge, UK). Membranes were washed with TBST and incubated for 1 h at room temperature with the secondary antibodies (Agilent-Dako, Santa Clara, CA, USA). After washing twice with TBST, bands were visualized with Super Signal (Pierce, Rockford, IL, USA) and developed with the Quantity One system. Optical density analysis of the bands was carried out with the Quantity One software. All proteins were normalized to the loading control beta-tubulin.

#### 4.3.3. Recordings of Intracellular Ca^2+^ and Patch Clamp Studies

The intracellular Ca^2+^ recordings were obtained from HL-1 cells transfected with miR-22-5p + miR-199a-5p or miR-NC and loaded with 5µM Fura-2AM (Molecular Probes, Eugene, OR, USA) during 45 min. Calcium measurements were obtained using an Olympus IX70 inverted microscope (Olympus, Hamburg, Germany). Cells were excited at 340 and 380 nm using a polychrome IV monochromator (Till Photonics, Munich, Germany) and fluorescence images were collected by a digital charge-coupled device camera (Hamamatsu Photonics, Japan) and viewed with the AquaCosmos software program (Hamamatsu Photonics, Japan). Throughout the experiments, cells were kept in an isotonic solution containing (in mM): 140 NaCl, 2.5 KCl, 1.2 CaCl_2_, 0.5 MgCl_2_, 5 glucose and 10 HEPES, pH 7.3 and 300–310 mOsmol/L. Intracellular calcium levels were recorded in the following conditions: basal resting conditions, following 10 mM of caffeine and after applying a hyperpolarizing solution containing (in mM): 60 NaCl, 75 KCl, 1.2 MgCl_2_, 5 CaCl_2_, 10 glucose and 10 HEPES, pH 7.3 and 300–310 mOsmol/L.

Cytosolic Ca^2+^ levels are graphed as the ratio of emitted fluorescence (510 nm) after excitation at 340 and 380 nm relative to the baseline. 340/380 nm ratio images were obtained every 5 s. Calcium decay post-caffeine stimulation was inferred by calculating the time constant Tau with the use of a script written for this paper.

Ba^2+^ currents through Cav1.2 channels were recorded miRNA-transfected HL-1 cells. Currents obtained by patch-clamp technique in a whole-cell configuration were recorded with a D-6100 Darmstadt amplifier (List Medical, Germany). Three independent transfections were patched. Pipettes had a resistance of 2-3 MΩ when filled with a solution containing (in mM): 140 CsCl, 1 EGTA, 4 Na_2_ATP, 0.1 Na_3_GTP and 10 HEPES, pH 7.2–7.3 and 290–300 mOsmol/L. The external solution contained (in mM): 140 tetraethylammonium-Cl (TEA-Cl), 2.5 BaCl_2_, 1.2 MgCl_2_, 10 glucose and 10 HEPES, pH 7.4 and 300–310 mOsmol/L. pClamp8 software (Molecular Devices, San Jose, CA, USA) was used for pulse generation, data acquisition and subsequent analysis. Membrane potential was kept at −30 mV and maximal peak IBa were recorded at +20 mV for 500 ms.

#### 4.3.4. Gap-Junction Monitoring

Gap-junctions were monitored by the diffusion of a fluorescent dye between attached cell pairs, as previously described [42]. HL-1 cells transfected with miR-22-5p + miR-199a-5p or miR-NC were seeded at very low confluences in gelatin-coated 12 mm diameter glass coverslips and incubated at 37 °C for 24 h in supplemented Claycomb medium. Afterwards, coverslips were mounted on a Zeiss Axiovert 10 microscope (Zeiss, Jena, Germany. Only attached cell pairs were selected, and one cell was injected with 0.1 pL of the molecule Alexa Fluor 488 (Alexa 570 MW, ThermoFisher, Waltham, MA, USA) using a FemtoJet 4i electric microinjector (Eppendorf, Hamburg, Germany). Images were acquired at 5 min intervals over a 90 min period to monitor the diffusion of the fluorescent dye from the injected cell to the adjacent one. These images were collected using a digital camera ZEISS Axiocam MRc and operated by the imaging software Zen (ZEISS, Jena, Germany).

### 4.4. Statistical Analyses

Selection of patients following the predefined matching criteria was performed with the statistical R software. Clinical data are reported as mean (SD) or frequency (%) as appropriate. MiRNA expression results are expressed with the 2^−^^ΔΔCt^ method [43]. Comparisons between groups were performed with Student’s *t*-test or chi-square analysis. For big-data analysis of OpenArray^®^ determinations, the specific CLOUD Software from Life Technologies (ThermoFisher, Waltham, MA, USA) was used to detect miRNAs differentially expressed among groups, with the mean global normalization approach. TargetScanHuman7.2 software was used to predict genes that could be potential targets of the miRNAs of interest. Statistical analyses were performed using the Statistical Package for Social Sciences 23.1 software package (SPSS Inc.) For all analyses in the study, differences were considered significant when *p* < 0.05.

## 5. Conclusions

This work demonstrates that 199a-5p and miR-22-5p are increased in plasma of HFrEF patients with AF compared to those in SR, and in atrial tissue obtained from patients with AF. Overexpression of both miRNAs in cultured atrial cells induces pro-arrhythmogenic responses. The potential role of high circulating levels of miR-199a-5p and miR-22-5p as biomarkers of AF in other contexts should be addressed in future studies.

## Figures and Tables

**Table 1:** Characteristics of the replication cohort.

	permAF (*n* = 40)	SR (*n* = 40)	*p*-Value
**Patient Characteristics**			
Sex, men (%)	31 (77.5)	31 (77.5)	NS
Age, y (SD)	73.2 (10.1)	73.5 (10.3)	NS
BMI, kg/m^2^ (SD)	30.5 (6.8)	27.5 (3.7)	0.02
**Medical history**			
Tobacco history (%)	22 (55)	22 (55)	NS
Hypertension (%)	30 (75)	31 (77.5)	NS
Diabetes (%)	14 (35)	18 (45)	NS
Hypercholesterolemia (%)	20 (50)	21 (52.5)	NS
COPD (%)	6 (15)	11 (27.5)	NS
CKD (%)	11 (27.5)	8 (20.0)	NS
Previous stroke (%)	8 (20.0)	4 (10.0)	NS
**HF parameters**			
Ischemic etiology (%)	16 (40)	24 (60)	NS
Heart rate, bpm (SD)	76.5 (16.6)	70.0 (14.6)	NS
NYHA class (%):			NS
I-II	16 (51.6)	24 (75)	
III-IV	15 (48.4)	8 (25)	
LVEF, % (SD)	32.5 (6.7)	32.8 (6.5)	NS
Log Pro-BNP, mean (SD)	3.4 (0.4)	3.3 (0.6)	NS
**HF treatment**			
ACEI/ARB (%)	34 (85.0)	33 (82.5)	NS
Betablockers (%)	37 (92.5)	37 (92.5)	NS
Diuretics (%)	40 (100)	37 (92.5)	NS
**Heart rhythm**			<0.001
Permanent SR	0	40	
History of parox/pers AF *	0	0	
Permanent AF ^#^	40	0	

BMI: body mass index; COPD: chronic obstructive pulmonary disease; CKD: chronic kidney disease; NYHA: New York Heart Association; LVEF: left ventricular ejection fraction; ACEI: angiotensin-converting-enzyme inhibitors; ARB: angiotensin-receptor blockers; SR: sinus rhythm; AF: atrial fibrillation. * Includes episodes of paroxysmal or short-standing persistent AF; ^#^ includes long-standing (>1 year) persistent or permanent AF. NS: non-significant.

**Table 2:** Results of the replication study.

	2^−^^△△Ct^ (permAF vs. SR)	*p*-Value
miR-106a-5p	1.292	0.378
miR-106b-5p	1.311	0.275
miR-125a-5p	1.395	0.110
miR-126-5p	1.244	0.258
miR-133a-3p	1.554	0.073
miR-16-5p	0.955	0.783
miR-17-5p	1.230	0.283
**miR-199a-5p**	**1.921**	0.028 *
miR-19a-3p	1.526	0.117
miR-20a-5p	1.158	0.452
**miR-22-5p**	**1.549**	0.033 *
miR-23a-3p	1.278	0.219
miR-26a-5p	1.322	0.187
miR-27b-3p	1.402	0.153
miR-301a-3p	1.115	0.765
miR-324-5p	1.531	0.088
miR-374a-5p	1.239	0.433
miR-425-3p	1.450	0.119

* Significant *p*-value < 0.05.

## Data Availability

The data presented in this study are available in this paper and supplementary file.

## Supplementary Materials

The following are available online at https://www.mdpi.com/article/10.3390/ijms221910377/s1.
