# Supplementary material for: Atrial Fibrillation in Heart Failure Is Associated with High Levels of Circulating microRNA-199a-5p and 22–5p and a Defective Regulation of Intracellular Calcium and Cell-to-Cell Communication
Source: Int J Mol Sci. 2021 Sep 26;22(19):10377. doi: 10.3390/ijms221910377 (PMC8508749; doi:10.3390/ijms221910377)
Supplement: Supplementary file 1 [file ijms-22-10377-s001.zip › ijms-1382724-supplementary.pdf]

International Journal of *Molecular Sciences*

MDPI

Article

Atrial Fibrillation in Heart Failure Is Associated With High Levels of Circulating microRNA-199a-5p and 22–5p and a Defective Regulation of Intracellular Calcium and Cell-to-Cell Communication

Anna Garcia-Elias [1,2,*], Marta Tajes [2,3], Laia Yañez-Bisbe [1,2], Cristina Enjuanes [3,4], Josep Comín-Colet [3,4,5], Selma A. Serra [6], José M. Fernández-Fernández [6], Kathryn W. Aguilar-Agon [7], Svetlana Reilly [7], Julio Martí-Almor [2,8,9] and Begoña Benito [1,2,9,10,11,*]

SUPLEMENTARY TABLES

Table S1. Characteristics of the first cohort.

	**permAF (n = 9)**	**SR (n = 9)**	***p*-Value**
Patient Characteristics			
Sex, men (%)	5 (55.5)	5 (55.5)	NS
Age, y (SD)	74.9 (8.2)	75.2 (7.0)	NS
BMI, kg/m^2 (SD)	26.8 (2.6)	26.8 (2.8)	NS
Medical history			
Tobacco history (%)	4 (44.4)	5 (55.5)	NS
Hypertension (%)	7 (77.8)	7 (77.8)	NS
Diabetes (%)	2 (22.2)	6 (66.7)	NS
Hypercholesterolemia (%)	4 (44.4)	7 (77.8)	NS
COPD (%)	2 (22.2)	1 (11.1)	NS
CKD (%)	2 (22.2)	2 (22.2)	NS
Previous stroke (%)	2 (22.2)	2 (22.2)	NS
HF parameters			
Ischemic etiology (%)	4 (44.4)	4 (44.4)	NS
Heart rate, bpm (SD)	70.2 (13.4)	80.0 (16.3)	NS
NYHA class (%):			NS
- I-II	6	6	
- III-IV	3	3	
LVEF, % (SD)	32.0 (6.5)	28.1 (5.6)	NS
Log Pro-BNP, mean (SD)	3.4 (0.5)	3.2 (0.6)	NS
HF treatment			
ACEI/ARB (%)	8 (88.9)	8 (88.9)	NS
Betablockers (%)	9 (100)	9 (100)	NS
Diuretics (%)	9 (100)	9 (100)	NS
Heart rhythm			<0.001
Permanent SR	0	9	
History of parox/pers AF*	0	0	
Permanent AF#	9	0	

BMI: body mass index; COPD: chronic obstructive pulmonary disease; CKD: chronic kidney disease; NYHA: New York Heart Association; LVEF: left ventricular ejection fraction; ACEI: angiotensin-converting-enzyme inhibitors; ARB: angiotensin-receptor blockers; SR: sinus rhythm; AF: atrial fibrillation. *Includes episodes of paroxysmal or short-standing persistent AF; #includes long-standing (> 1 year) persistent or permanent AF. NS: non-significant.

Table S2. MicroRNAs differentially expressed in the discovery phase.

miRNA	*p*-Value	miRNA	*p*-Value	miRNA	*p*-Value
miR-505#	0.000701	miR-1	0.010444	miR-140	0.024151
miR-20a	0.002003	miR-151-5P	0.010454	miR-671-3p	0.024503
miR-539	0.002037	miR-645	0.011216	miR-664	0,.024804
**miR-425#**	**0.002114**	miR-7#	0.011294	miR-195	0.025214
**miR-106a**	**0.002469**	miR-93#	0.011334	miR-9#	0.025847
miR-30d#	0.002633	miR-130b#	0.012461	miR-124a	0.026115
**miR-19a**	**0.004109**	miR-186	0.012493	miR-495	0.026444
**miR-16**	**0.004259**	miR-652	0.012506	miR-151-3p	0.026993
miR-628-3p	0.004991	**miR-26a**	**0.012758**	**miR-106b**	**0.029477**
**miR-199a-5p**	**0.006002**	miR-432	0.014040	miR-340	0.030723
**miR-301**	**0.006242**	let-7d	0.014543	miR-185	0.033889
**miR-374**	**0.006285**	**miR-23a**	**0.014962**	miR-411	0.035389
**miR-133a**	**0.006676**	miR-636	0.015891	miR-590-3P	0.035525
miR-374-5p	0.007119	**miR-20a#**	**0.016181**	miR-98	0.035920
miR-330	0.007449	**miR-324-5p**	**0.016251**	miR-30e-3p	0.035936
**miR-126#**	**0.007465**	miR-191#	0.016514	miR-1180	0.036382
**miR-17**	**0.007740**	miR-140-3p	0.016646	miR-27a	0.037929
miR-142-5p	0.007847	miR-625	0.017292	miR-331-5p	0.040697
miR-1255B	0.008360	miR-483-3p	0.017459	miR-656	0.042341
miR-598	0.008435	miR-744	0.018172	miR-107	0.043088
miR-26a-1#	0.008641	miR-489	0.018316	miR-331	0.043250
**miR-27b**	**0.008667**	miR-24	0.019166	miR-326	0.043429
**miR-22-5p**	**0.008810**	miR-28	0.019784	miR-146b	0.043554
miR-148b	0.009361	miR-380-3p	0.021854	miR-221	0.046365
miR-191	0.010162	miR-580	0.023425	miR-15b	0.048038
**miR-125a-5p**	**0.010217**				

MiRNA selected for the replication phase are marked in bold.

Table S3. Characteristics of the donors of atrial tissue.

Tissue Collection Site	LAA		RAA	
	**No AF**	**AF**	**No AF**	**AF**
Total number of patients	6	6	5	5
Mean age, years (± SD)	60 ± 14	66 ± 12	64 ± 10	68 ± 12
Men, *n* (%)	4 (67)	5 (83)	4 (80)	5 (100)
Surgical procedure, n (%)				
CABG ± AVR/MVR	2 (33)	1 (17)	2 (40)	1 (20)
AVR/MVR	4 (67)	5 (83)	3 (60)	4 (80)
Medical history, n (%)				
Smoker/ex-smoker	4 (67)	2 (33)	3 (60)	1 (20)
Hypertension	3 (50)	3 (50)	2 (40)	3 (60)
Diabetes mellitus	2 (33)	0 (0)	0 (0)	0 (0)
Previous MI	1 (17)	2 (33)	0 (0)	0 (0)
COPD/asthma	0 (0)	0 (0)	0 (0)	0 (0)
Medications, n (%)				
Anticoagulants	1 (17)	4 (67)	0 (0)	3 (60)
β-Blockers	2 (33)	5 (83)	3 (60)	5 (100)
Statins	2 (33)	3 (50)	1 (20)	3 (60)
Calcium-channel blockers	0 (0)	0 (0)	0 (0)	1 (20)
ACEIs and ARBs	1 (17)	4 (67)	1 (20)	3 (60)
Diuretics	0 (0)	2 (33)	2 (40)	2 (40)

LAA, left atrial appendage; RAA, right atrial appendage; noAF, sinus rhythm; AF, atrial fibrillation; ACEI, angiotensin-converting enzyme inhibitor; ARB, angiotensin II receptor blocker; AVR, aortic valve replacement; CABG, coronary artery bypass surgery; COPD, chronic obstructive pulmonary disease; MI, myocardial infarction; MVR, mitral valve replacement. Percentage in parentheses (%) indicates percentage within the same group.

Table S4. MiRNA primer sequences for atrial tissue.

No AF	AF

Assay Name	Assay ID	Target Sequence 5′-3′
hsa-miR-22-5p	477987_mir	AGUUCUUCAGUGGCAAGCUUUA
hsa-miR-199a-5p	478231_mir	CCCAGUGUUCAGACUACCUGUUC
hsa-miR-191-5p	477952_mir	CAACGGAAUCCCAAAAGCAGCUG
hsa-miR-26a-5p	477995_mir	UUCAAGUAAUCCAGGAUAGGCU

SUPLEMENTARY FIGURES

FIG.S1

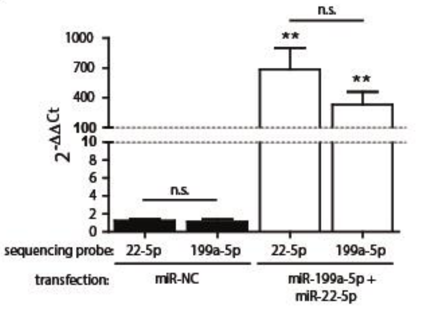

FIG.S2

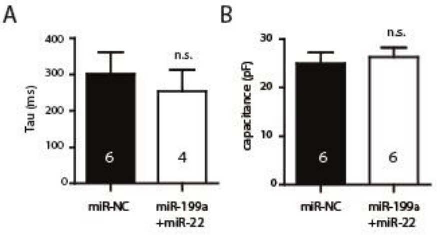

FIG.S3

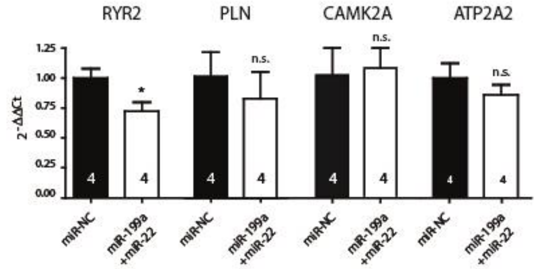

Figure S1. RNA expression levels of miR-22-5p and miR-199a-5p in HL1 cells transfected with miR-199a+miR-22 or miR-NC. Specific probes for each miRNA were used. Data are expressed as the fold change ($2^{-\Delta\Delta Ct}$) relative to the negative control, miR-NC. **Figure S2**. A. Fast inactivation kinetics (Tau) of I_{CaL} currents and, B. cell capacitance of HL-1 cells transfected with miR-199a+miR-22 (white) or miR-NC (black). Numbers within the columns represent number of patched cells. **Figure S3**. *RYR2*, *PLN*, *CAMK2A* and *ATP2A2* mRNA expression levels in miR-199a+miR-22 HL-1 cells, normalized to miR-NC. *n*.s.: non-significant, * $p < 0.05$, ** $p < 0.005$.
